# Protective Effect of Ginkgolic Acid in Attenuating LDL Induced Inflammation Human Peripheral Blood Mononuclear Cells *via* Altering the NF-κB Signaling Pathway

**DOI:** 10.3389/fphar.2019.01241

**Published:** 2019-11-08

**Authors:** Juan Zhang, Jifeng Yan

**Affiliations:** ^1^Department of Cardiovascular Medicine, The Second Affiliated Hospital of Zhengzhou University, Zhengzhou, China; ^2^Heart Center of Henan Provincial People’s Hospital, Central China Fuwai Hospital, Zhengzhou, China; ^3^Central China Fuwai Hospital of ZhengZhou University, ZhengZhou, China

**Keywords:** ginkgolic acid, inflammation, prostaglandin E_2_, atherosclerosis, oxidized low-density lipoprotein

## Abstract

Oxidized low-density lipoprotein (ox-LDL) is considered as the significant maker of inflammatory reaction. ox-LDL was reported to play a crucial role in the pathogenesis of atherosclerosis (AS). In the current study, we scrutinize the suppressive effect of ginkgolic acid against ox-LDL induced an oxidative and inflammatory response in human microvascular endothelial cells (HMEC-1) and human peripheral blood mononuclear cells (nPBMCs) and explore the mechanism of action. HMEC-1 cells are treated with ox-LDL in the presence of different concentration of ginkgolic acid. MTT 3-(4,5-dimethylthiazol-2-yl)-2,5-diphenyltetrazolium bromide) assay was performed for the estimation of cell viability effect. Reactive oxygen species (ROS), inflammatory cytokines, and NF-κB activity are also estimated. For the hPBMCs assay, the cells were isolated from the healthy volunteers and cultured. The cells were further divided into different group and received the ginkgolic acid. Additionally, ROS, inflammatory marker such as prostaglandin E_2_ (PGE_2_), lipoxygenase (LOX), nitric oxide (NO), cyclooxygenase (COX) protein expression, and mRNA expression of tumor necrosis factor-α (TNF-α), interleukin-6 (IL-6), and vascular cell adhesion protein 1 (VCAM-1) were estimated in the ox-LDL treated group. The result exhibited that ginkgolic acid treatment induced the cell viability boosting in ox-LDL treatment and intracellular ROS significantly decreased by ginkgolic acid. Pro-inflammatory cytokines also downregulated *via* ginkgolic acid. Moreover, ginkgolic acid reduced the ox-LDL–induced NF-κB. The mRNA and protein expression of TNF-α, IL-6, and VCAM-1 considerably increased in the ox-LDL treated group and ginkgolic acid significantly reduced the mRNA and protein expression. An inflammatory marker such as PGE_2_, LOX, and NO were increased in the ox-LDL treated group and ginkgolic acid treated group exhibited the reduction of an inflammatory marker. Based on the result, we can conclude that ginkgolic acid significantly reduced and reversed the ox-LDL–induced modulation, suggesting its anti-inflammatory effect *via* the NF-κB pathway.

## Introduction

The researcher suggests that the oxidation of low lipid lipoproteins (ox-LDL) is considered as the important marker of inflammatory reaction. ox-LDL has been well known to boost the pro-inflammatory cytokines leading to monocyte infiltration into the vessel walls (Burstein et al., 1970; Adiels et al., 2008; Jonas and Phillips, 2008). Research suggests that the generation of ox-LDL during the oxidative stress condition enhanced the reactive oxygen species (ROS) production, which further gathers in macrophages and other cells and creates the chronic inflammatory condition (Aviram and Rosenblat, 2004; Gritters et al., 2006; Lerner et al., 2015). Ox-LDL also boosts the inflammatory reaction and oxidative stress to the vascular endothelium (Hartge et al., 2007; Vanhoutte et al., 2009). It also exerts the atherosclerotic plaque progression and formation and enhances the secretion and synthesis of adhesion molecules, adhesion, and monocyte chemotaxis (Gharavi et al., 2007; Vanhoutte et al., 2017); ox-LDL enhanced the smooth muscle cell proliferation and amplify foam cell formation, endothelial cell apoptosis, matrix degradation, and oozing of matrix metalloproteinases (MMP) arbitrating the degradation protein components of extracellular matrix and basement membrane in target cells (Suematsu et al., 2002; Gharavi et al., 2007; Potenza et al., 2008; Lu et al., 2011; Yung et al., 2012).

The researcher suggests that it also increased the overexpression of adhesion molecule on the endothelial cell surface and also enhanced the monocytes to the arterial wall of endothelial dysfunction (Kubes et al., 1991). Endothelial dysfunction has been considered as a significant factor for the progression of cardiovascular diseases. Endothelial dysfunction could induce platelet adhesion and cardiovascular inflammation (CVD) especially atherosclerosis (AS) (Cai and Harrison, 2000; Steyers and Miller, 2014; Papadimitraki and Boumpas, 2015). The report suggests that the CVD majorly leading the cause of mortality and morbidity worldwide and approximately 16 million death has been reported annually (Hadi et al., 2005; Higashi et al., 2009; Gimbrone and García-Cardeña, 2016). CVD disease, mainly AS is a chronic disease of the arterial vessel wall, started *via* deposition of lipoproteins in the intimal layer of the vascular wall (Reis and Lutsey, 2012; Ho, 2018; Reamy et al., 2018). AS is responsible for a large number of casualties related to cardiovascular-related disease (Mendis et al., 2011; Troosters, 2012). AS is a chronic and complex inflammatory disease, which is described by the abnormal deposition of lipids and fibrous elements into the arteries (Holligan et al., 2012). It is frequently asymptomatic for several decades until the incidence of serve CVD such as heart attack or stroke (Holligan et al., 2012). Oxidized lipids are responsible for the onset of expression of a set of genes that cause the chronic inflammatory reaction, leading to the deposition of oxidized lipids within the vessel wall (Friedewald et al., 1972; Hamilton, 1997). Due to deposition of oxidized lipids inside, the vessel wall was able to increase the expression of transcription factors genes, *viz*., nuclear factor κB (NF-κB), to induce the chronic inflammatory reaction (Dichtl et al., 1999; Boersma et al., 2011). Research suggests that the inflammatory process involved in every step of AS starts from the damaging in a vessel of endothelial cells to burst of plaque at the end-stage (Slager et al., 2005; Lamon and Hajjar, 2008). During the progression of AS, the formation of foam cells (lipid-laden macrophages) plays a significant role in the retort to inflammation linked stimuli (Esper et al., 2008; Lamon and Hajjar, 2008). Usually, the formation of the above disease is related to the hyperlipidemia and unusual deposition of ox-LDL (Akpolat et al., 2011; Hlaing and Park, 2013). Research suggests that the ox-LDL damaged the endothelial function resultant, increased the production of ROS, and reduced the production of nitric oxide (NO) (Rao, 2002; Mitra et al., 2011a). Additionally, continue generation of pro-inflammatory reaction could activate the macrophages to produce ROS, which induces the apoptosis and involved in the subsequent plaque formation in the progression of AS progression. Lipooxygenase-1 (LOX-1) plays an important role in the boosting of inflammatory reaction (Negre-Salvayre et al., 2006; Mitra et al., 2011a). The uptake of ox-LDL into the endothelium interacts with LOX-1 receptor and induces the toxic side effect such as generation of ROS, secretion of pro-inflammatory cytokines, proapoptotic proteins, and overproduction of adhesion molecules (Brucker et al., 2013; Ding et al., 2013). The nuclear level, central transcriptional (NF-κB) plays an important role in the activation of vascular endothelium during the atherosclerosis disease. Previous studies suggest that the NF-κB and generation of intracellular ROS both play a crucial role in the inflammatory reaction. Research suggests that the ox-LDL activates the NF-κB and augments the production of ROS in endothelial cells (Ross, 1999; Lluís et al., 2013). Toll-like receptor 4 (TLR4) plays a vital role in the regulation and initiation of the immune response and induces remarkable proatherogenic and pro-inflammatory cytokines expression in endothelial cells and macrophages. Adaptor molecule such as myeloid differentiation factor 88 (Myd88) plays a crucial role in the TLR4 signaling pathway. Another inflammatory pathway such as NF-κB p65 altered the TLR4 pathway, resultant induces the expression of various pro-inflammatory mediators, which contributes to the atherosclerosis disease (Geng et al., 2010; Yu et al., 2011; Rocha et al., 2016).

Pro-inflammatory cytokines, *viz*., interleukin-1 (IL-1) and interleukin-2 (IL-2), produced *via* macrophages exert inflammatory effects and induce the expansion of AS. It is believed that chronic inflammation plays an important factor in the progression of atherogenesis (Han et al., 2010; Yu et al., 2016). Another inflammatory marker, *viz*., TNF-α and IL-6, played a crucial role in ox-LDL–induced inflammation. IL-6 shows the direct effect inflammatory, proatherogenic effect including activation of endothelial cells and platelet activation, enhances the vascular smooth muscle proliferation, and increases the production of ROS (Schleicher and Friess, 2007; Yan et al., 2014; Han et al., 2016). Imbalance of cholesterol influx, efflux, and synthesis could interrupt the cholesterol homeostasis and start the generation of form cells. Due to the direct role of pro-inflammatory cytokines and alteration of intracellular ROS level, the researcher focuses on their research to treat the AS disease (Yan et al., 2014; Lin et al., 2017).

## Materials and Methods

### Chemical

**Figure 1:**
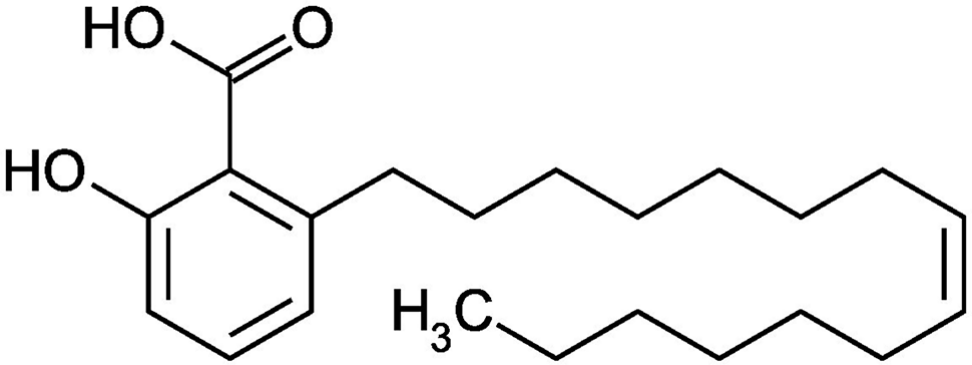
Structure of Ginkgolic acid.

Ginkgolic acid (≥95.0%) (**Figure 1**) was purchased from the Sigma Aldrich, USA.

### Cell Culture

Human microvascular endothelial cells (HMEC-1) were used for the current experimental study. The cells were cultured in the MCDB 131 medium containing fetal bovine serum (10%) and cells were cultured into the humidified air area containing CO2 at 37°C. The cells were treated with 200-μg oxidized LDL (ox-LDL) in the presence of ginkigolic acid (0–20 μM) for 1 day. After that, the biomarkers in the supernatant and cells were detected.

### Cell Viability Assay

The cells were seeded overnight in 96-well plates and, afterward, mixed the ox-LDL and various concentration of ginkgolic acid into the wells at 37°C for 1 day. On the other hand, the vehicle control group contain DMSO (0.5%). The MTT assay was used for the determination of cytotoxicity. Briefly, MTT (100 μl) was added into the medium and left for next 4 h. Consequently, isopropanol (150 μl) was added into the medium for the next 15 min and finally calculated the absorbance at 570 nM *via* using the microplate reader. Meanwhile, the result was presented as the relative ratio as compared with the vehicle group.

### Determination of ROS

Fluorescent probes DCFH2-DA was used for the estimation of intracellular ROS production *via* using the previous method with minor modification. The HMEC-1 cells were treated with the ox-LDL (200 μg) and ginkgolic acid, and subsequently, DCFH2-DA was added for 20 min at the temperature (37°C) in the dark place. For the estimation of intracellular ROS levels, fluorescence was used for excitation (488 nM) and emission (519 nM) *via* using the confocal microscope.

### Lipid Peroxidation (Lpo) Assay

For the estimation of LPO, malondialdehyde (MDA) production was estimated *via* using the previously reported method with minor modification (Wiseman et al., 2000; Ferretti et al., 2010). Briefly, 0.55-ml LDL was added in all tube and added the trichloroacetic acid (0.5%) to denature the protein. The sample was centrifuged at 10,000 rpm for 30 min at 10°C to separate the pellets. 0.5 ml of thiobarbituric acid (TBA) added in the supernatant and vigorously mixed the reagents to react for 40 min at 90–95°C in a dark room. After completion of the reaction, the absorbance was estimated at 532 nM (excitation) and 600 nM (emission).

### Relative Electrophoretic Mobility

For REM, 200 μg/ml of LDL was pretreated with the various concentration of ginkgolic acid for 2 h and followed incubation at 37°C with CuSO_4_ (10 μM) for 16 h. LDL was estimated *via* using the agarose electrophoresis to estimation the increase in electrophoretic mobility. Briefly, modified LDL was loaded into agarose gels (0.6%) and electrophoresed at 100 V for 40 min.

### ApoB Fragmentation

After the oxidation in the presence and absence of ginkgolic acid, samples were denatured with 2-mercaptoethanol (5%), SDS (3%), and glycerol (10%) for 5 min at 95°C for the detection of polyacrylamide gel electrophoresis.

### 2,2-Diphenyl-1-Picrylhydrazyl (DPPH) Radical Scavenging Method

For the estimation of the antiradical activity of ginkgolic acid, DPPH model was used *via* following the previously reported method with minor modification (Kumar et al., 2015). Briefly, DPPH (0.2 nM) is freshly prepared *via* dissolving the DPPH in the methanol solution. Then, ginkgolic acid and trolox were added into the DPPH solution and finally estimated the concentration at 517 nM after the incubation of the sample mixture in room temperature.

### Estimation of Mitochondrial Membrane

For the estimation, the effect of ginkgolic acid on the mitochondrial membrane has a potential *via* using the lipophilic cationic probe fluorochrome5,58,6,68-tetraethylbenzimidazolcarbocyanine iodide (JC-1) (Cossarizza et al., 1993; Macho et al., 1996). JC-1 shows the potential dependent deposition in mitochondria as an indicator of fluorescence emission. After the treatment of ox-LDL for 16h with and without treatment of ginkgolic acid, the cells were washed with medium followed *via* the addition of JC-1, and finally, the cells were scrutinized *via* using the fluorescent microscope.

### Pro-Inflammatory Cytokines Estimation

For the estimation of inflammatory cytokines, the supernatants of HMEC-1 cells were collected after the ox-LDL and ginkgolic acid treatment. Pro-inflammatory cytokines such as TNF-α and IL-6 were quantified in the HMEC-1 cells *via* using the enzyme-linked immunosorbent assay (ELISA; R and D Systems, Minneapolis, MN, USA) by following the manufacturer’s instruction.

### Estimation of NF-κB Production

After the ox-LDL and ginkgolic acid treated HMEC-1 cells, the nuclear extracts from the treated cells were prepared *via* using the Nuclear Extract Kit. NF-κB p65 assay kit was used for the estimation of NF-κB activity *via* using the (SN368, Beyotime Institute of Biotechnology, China) manufacture’s instruction.

### Preclinical Study

#### Preparation of LDL

For the collection of LDLs, the blood sample was withdrawn from the overnight fasted donor and kept in precooled vacuum tubes containing Na-EDTA. For the separation of the plasma, the samples were centrifuged at low speed at 1°C and kept the same temperature throughout the separation protocol. The LDL was successfully isolated from the plasma in the density interval of 1.025 to 1.050 kg/l by sequential preparative ultracentrifugation for 20. Lowry technique was used for the estimation of protein level in the LDL preparation.

#### Oxidation of LDL *via* Copper

Firstly, separate the EDTA from the isolated LDL *via* dialyzed in 0.02 mol/L phosphate/0.16 mol/L NaCl buffer, pH 7.4 for 15 h at low temperature (4°C). Copper-mediated oxidation of LDL was performed *via* incubating the EDTA-free LDL (0.2 mg/ml) in medium containing CuSO4 (10-5 mol/L) at 37°C for 12 h. Limulus assay was used for the analysis of presence or absence of endotoxins in the LDL preparations. In the whole procedure, the endotoxin levels should be less than 0.5 ng/ml in the stock solutions and less than 5 pg/ml in the test samples. The thiobarbituric acid reactive substance was used for the estimation of lipid peroxidize content in the native and oxidized LDL.

#### Isolation of Human Peripheral Blood Mononuclear Cells

For the isolation of human peripheral blood mononuclear cells, briefly, the isolated cells were cultured in collagen I coated plates and cultured in RPMI medium. The cells were dispersed in culture plates and incubated and different concentration of ginkgolic acid for 24 h. We further divided the groups as follows: Gp- I control, Gp II- ox-LDL, Gp-III ox-LDL+GA (5 μg/ml) and Gp-IV ox-LDL+GA (20 μg/ml). Furthermore, the cells were used for the estimation of lipoxygenase (LOX), cyclooxygenase (COX), prostaglandin E2 (PGE2), nitric oxide (NO), interleukin-6 (IL-6), tumor necrosis factor-α (TNF-α), vascular cell adhesion molecule-1 (VCAM-1), nuclear factor-kappa B (NF-κB), and toll-like receptor 4 (TLR4) after the incubation (24 h).

#### Cytotoxicity and NF-κB Transcription Assay

Tetrazolium salt 3-(4-5-dimethylthiozol-2-yl) 2-5-diphenyl-tetrazolium bromide (MTT) assay was used for the estimation of cytotoxicity effect *via* using the previous method with minor modification.

NF-κB p65 nuclear translocation was estimated by using the standard kits *via* following the manufacturer’s instruction (M/s Cayman chemicals, MI, USA).

#### Estimation of Inflammatory Markers

The inflammatory markers such as COX and LOX were estimated *via* using the previously published method with minor modification. NO activity was determined by using the previously published protocol of Kumar et al., with minor modification.

For the estimation of PGE2, available ELISA kits were used *via* the following manufacturer’s instruction (M/s Cayman chemicals, MI, USA).

#### Western Blot Technique

Various parameters such as IL-6, VCAM-1, and TNF-α were estimated *via* using the western blot techniques. hPBMCs were washed with phosphate buffer saline (PBS) three times before the lysis on ice *via* using the 10 mmol/EDTA, 150 MMOL/NaCl, 10 mmol/Tris, 10 mmol/NaN3, 5 mmol/iodoacetamide, 1% NP-40, and 1 mmol/PMSF. Finally, the lysates were heated for 5 min in nonreducing sample buffer [2% SDS, 0.001% bromophenol blue, 10 mmol/Tris (pH 6.8), 20% glycerol, and resolved *via* 8% SDS–polyacrylamide gel electrophoresis (PAGE)]. The separated protein was successfully moved to the nitrocellulose membrane and blocked at room temperature for 1 h, and finally, the membrane was incubated with specific antibodies against primary antibodies for overnight at 4°C followed *via* conjugated with secondary antibodies for 60 min at 37°C. Diaminobenzidine substrate solution was used for the detection of antibodies.

#### RT-PCR Analysis

RNA isolation kits were used for the isolation of total cellular RNA *via* following the manufacturer’s instruction, and finally, the UV spectroscopy was used for the quantification of isolated RNA *via* estimated the absorbance at 260 and 280 nM. The cDNA first strand was synthesized using the total RNA in the Eppendorf thermal cycler and the primer was added in the reaction mixture in the presence of dNTPs, reverse transcriptase, and RNase inhibitor. The reaction mixture was gently centrifuged for 5 min (25°C) followed *via* 1 *h* (42°C) for reverse transcription, and finally, the reaction was terminated *via* heating the reaction mixture for 5 min at 70°C. The sequences of the primers are presented in **Table 1**.

**Table 1:** Showed the list of primers.

S. No	Gene	Forward	Reverse
1	TLR-4	5' GCAGAAAATGCCAGGATGATG3'	5'GGCTGTCAGAGCCTCGTGGCTT TGG3'
2	TNF-α	5' GCA GAA AAT GCCAGG ATG ATG3'	5'GGC TGT CAG AGC CTC GTG GCT TTG G3'
3	iNOS	5'CAG CAC AGA GGG CTCAAA GC3'	5'TCG TCG GCC AGC TCTTTC T3'
4	VCAM-1	5'-AAA AGC GGA GACAGG AGA CA-3'	5'-AGC ACG AGA AGCTCA GGA GA3'
5	IL-6	5'CCA CTG CCT TCC CTACTT CA3'	5'TGG TCC TTA GCC ACTCCT TC3'
6	GAPDH	5'TGAAGGTCGGTGTGAACGGATTT GGC3'	5'CATGTAGGCCATGAGGTCCACC AC3'

### Statistical Analysis

For the statistical analysis, one-way ANOVA was used to identify the comparison between the different groups. Tukey test was used for the one-way ANOVA. Results are given as mean ± standard error of the mean.

## Result

### Effect of Ginkgolic Acid on HMEC-1 Cell Viability

**Figure 2:**
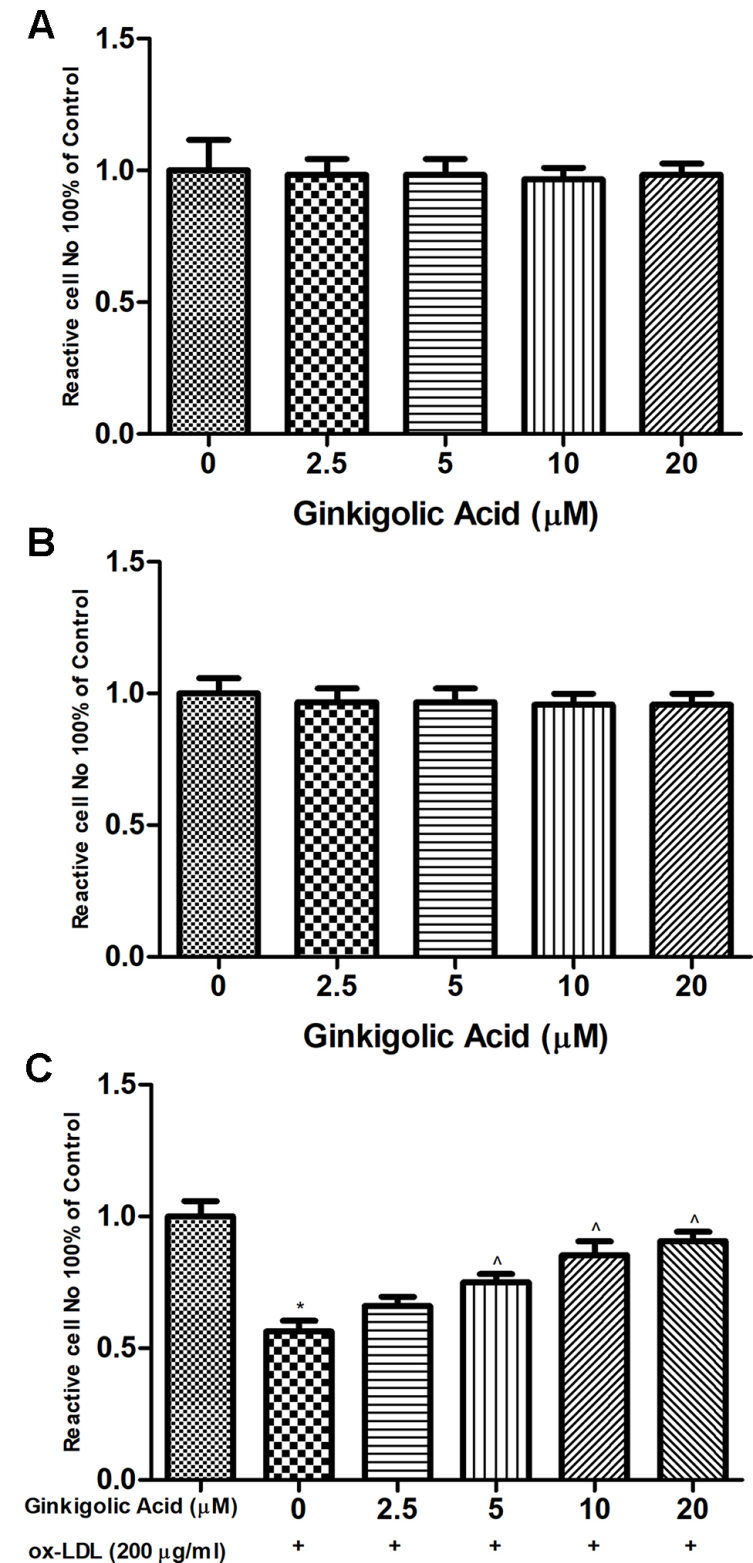
Showed the effect of ginkigolic acid on ox-LDL decreases HMEC-1 cell expansions indentify by MTT assay for **(A**, **B)** 24 and 48 h, **(C)** the cells (HMEC-1) treated with ginkigolic acid. Data are presented as Mean ± SEM, *p < 0.05, compared to the control group (without treatment), ^p < 0.05, compared to ox-LDL group.

MTT assay was performed for the estimation of a cytotoxicity study of ginkgolic acid on the HMEC-1 cell. For the MTT assay, 0–20 µM ginkgolic acid to choose for optimum dose to avoid cytotoxicity. MTT assay showed that the ginkgolic acid (5–20 µM) did not show any effect on the HMEC cell viability (**Figures 2A, B**). **Figure 2C** showed that the ginkgolic acid increased the cell viability in the ox-LDL–induced HMEC-1 cells.

### Effect of Ginkgolic Acid on Oxidative Stress

**Figure 3:**
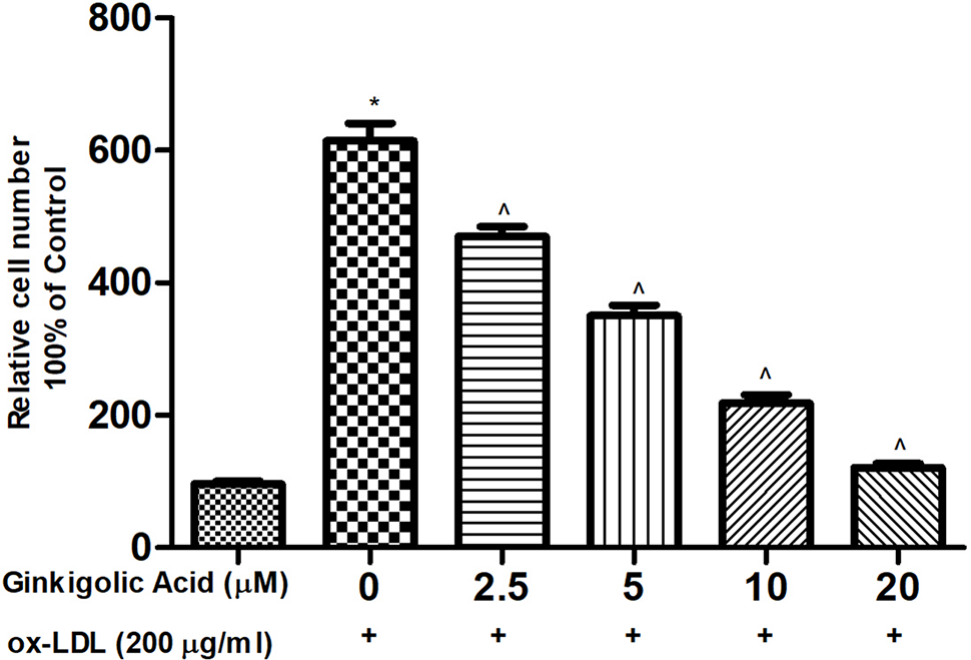
Showed the effect of ginkigolic acid on ox-LDL–induced ROS level. Data are presented as Mean ± SEM, *p < 0.05, compared to the control group (without treatment), ^p < 0.05, compared to ox-LDL group.

**Figure 3** showed the effect of ginkgolic acid on the intracellular ROS. For the estimation of oxidative stress, intracellular ROS were determined. The HMEC-1 cells treated with ox-LDL exhibited the increased intracellular ROS level. Ginkgolic acid treated HMEC-1 cell exhibited a reduced level of intracellular ROS in a concentration-dependent manner.

### Effect of Ginkgolic Acid on Inflammatory Cytokines

**Figure 4:**
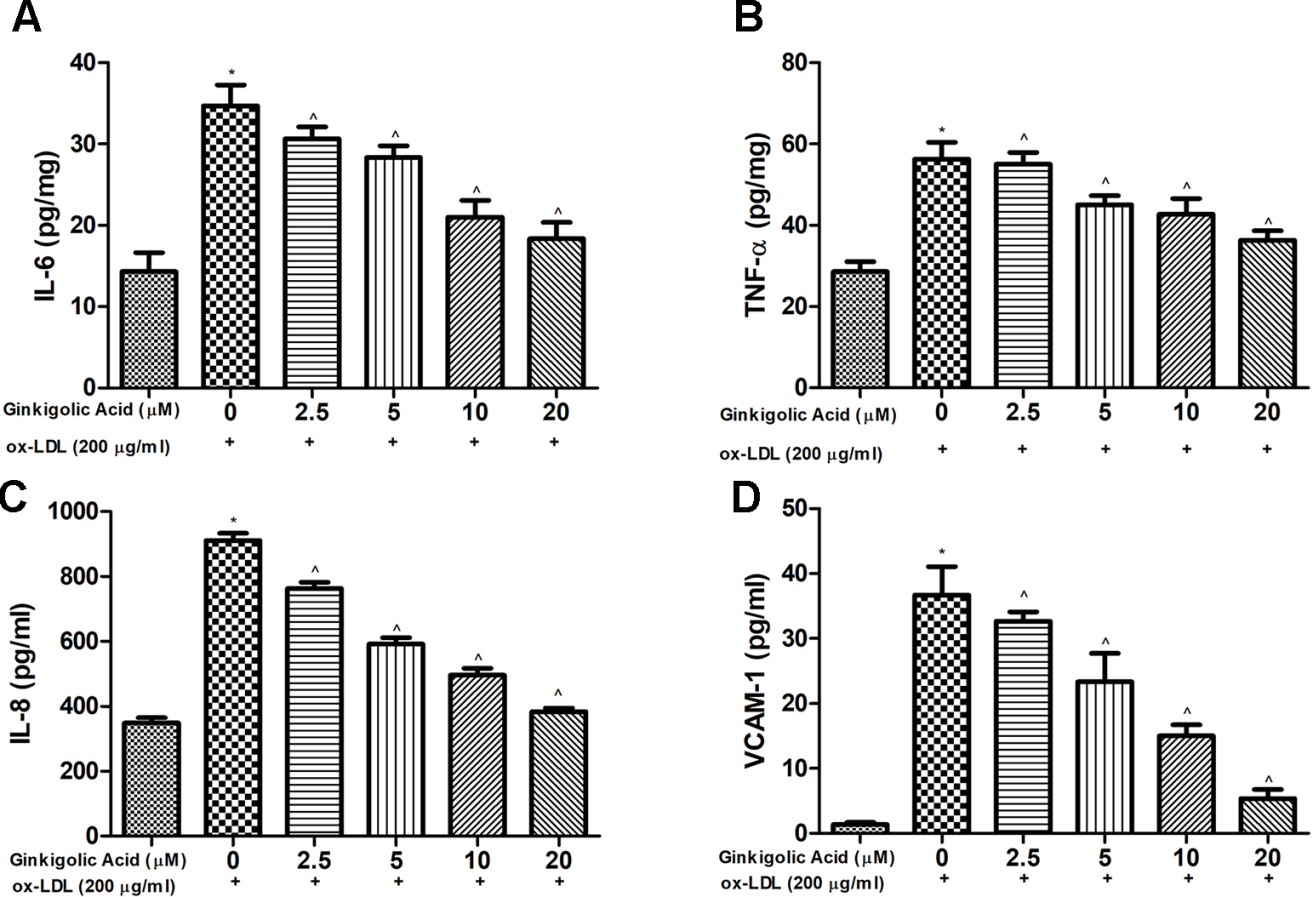
Showed the effect of ginkigolic acid on pro-inflammatory cytokines. **(A)** IL-6, **(B)** TNF-α and **(C)** IL-8 and **(D)** VACM-1. Data are presented as Mean ± SEM, *p < 0.05, compared to the control group (without treatment), ^p < 0.05, compared to ox-LDL group.

For the estimation of the anti-inflammatory effect of ginkgolic acid, pro-inflammatory cytokines were estimated. Ox-LDL treated cells showed the increased level of pro-inflammatory cytokines such as IL-6, TNF-α, IL-8, and VCAM-1, and concentration-dependent treatment of ginkgolic acid significantly reduced the level of pro-inflammatory cytokines (**Figure 4**).

### Effect of Ginkgolic Acid on Caspase and NF-κb

**Figure 5:**
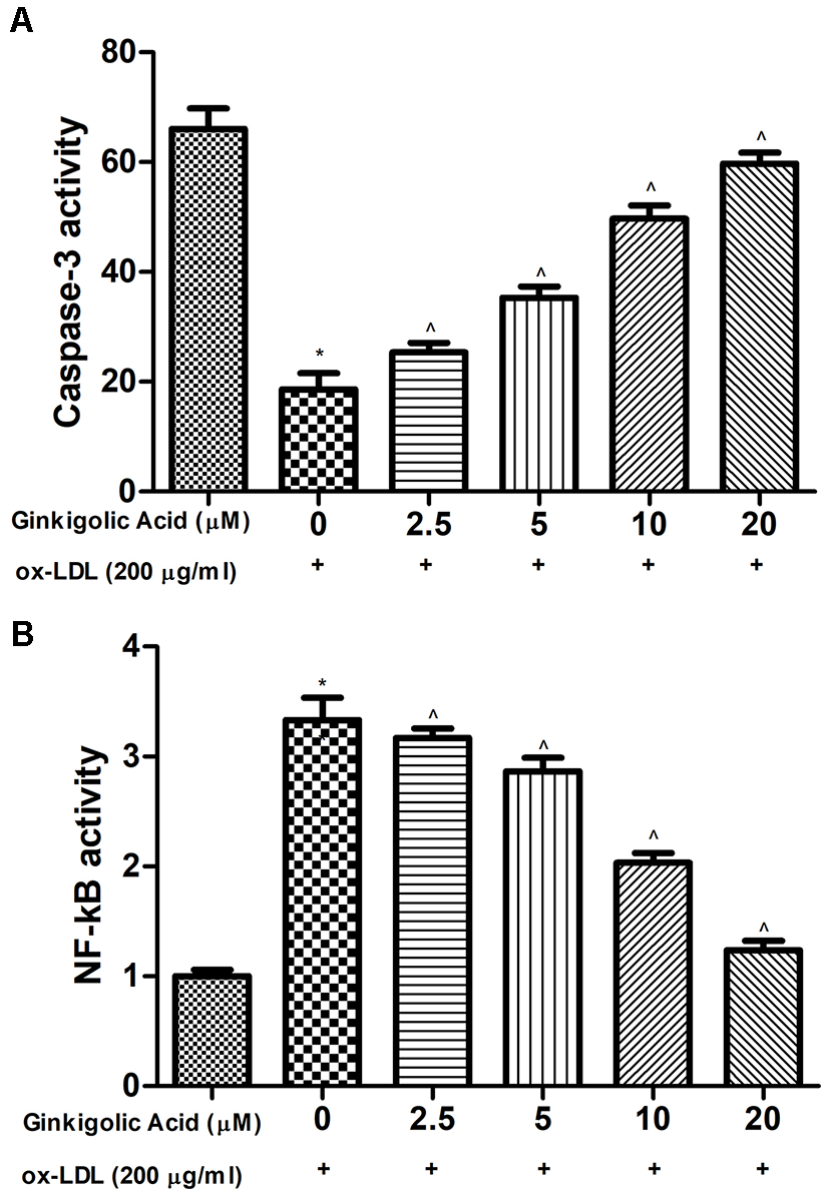
Effect of ginkgolic acid on the caspase-3 and NF-κB activity on the ox-LDL treated group. **(A)** caspase-3 and **(B)** NF-κB activity. Data are presented as Mean ± SEM, *p < 0.05, compared to the control group (without treatment), ^p < 0.05, compared to ox-LDL group.

**Figure 5** showed the effect on the caspase-3 and NF-κB in the ox-LDL and ginkgolic acid treated group. Ox-LDL treated group exhibited a reduced level of caspase-3 activity and increased the level of NF-κB activity. Ginkgolic acid significantly increased the caspase-3 activity and reduced NF-κB activity.

### Effect of Ginkgolic Acid on the Inflammatory Marker in hPBMCs

**Figure 6:**
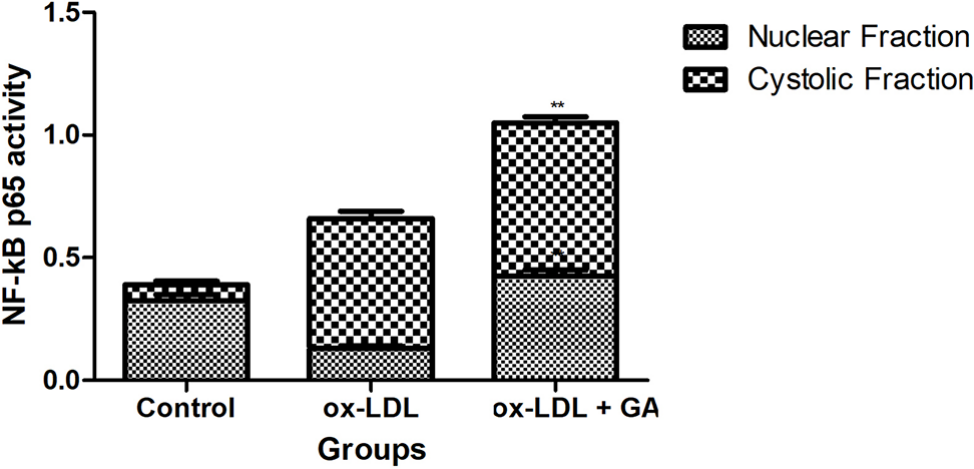
Showed the effect of ginkgolic acid on the NF-kB p65 Activity. Data are presented as Mean ± SEM, *p < 0.05, compared to ox-LDL group.

**Figure 6** exhibited the effect of ginkgolic acid on the inflammatory mediator. Ox-LDL group exhibited the increased level of LOX, NO, and PGE_2_ and dose-dependent treatment of ginkgolic acid significantly reduced the level of LOX, NO, and PGE_2_.

### Effect of Ginkgolic Acid on IL-6, TNF-α, and VCAM-1 in hPBMCs

**Figure 7:**
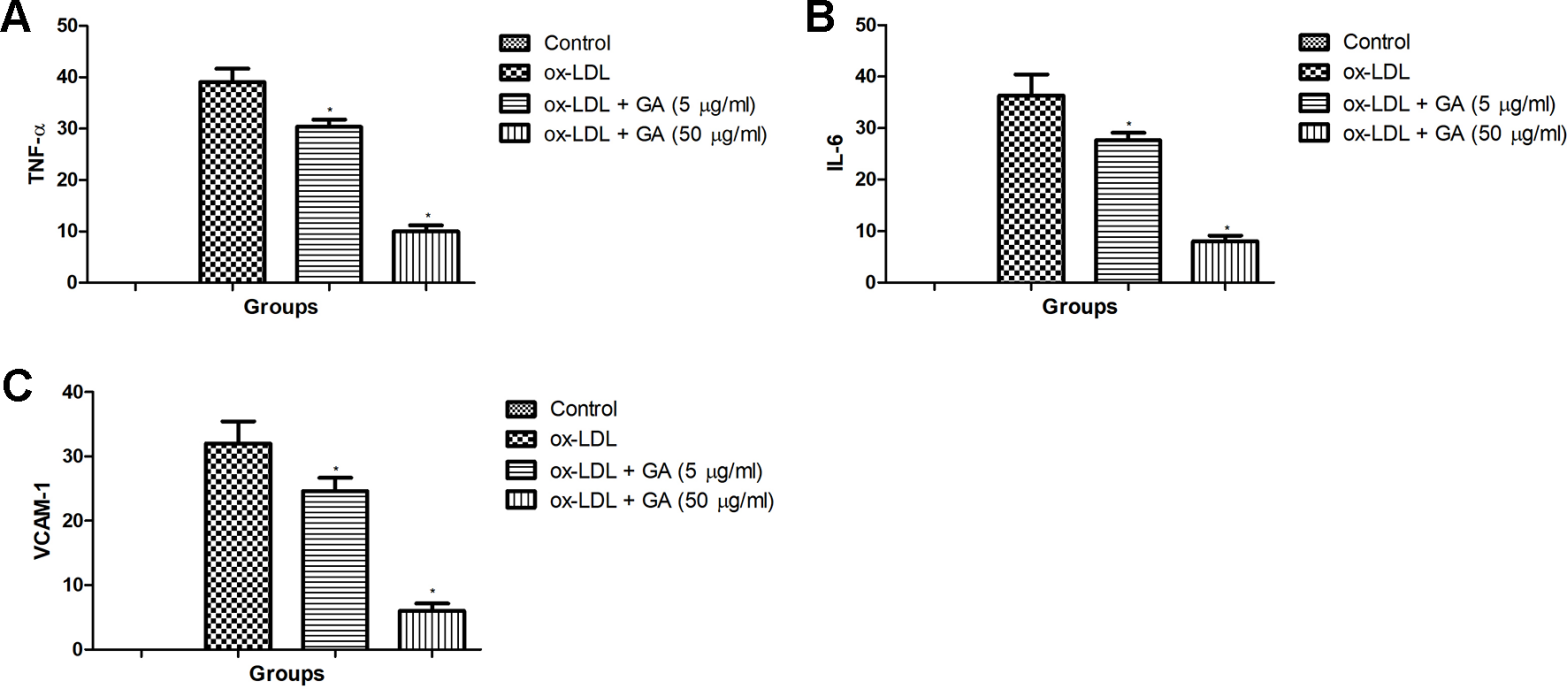
Showed the effect of ginkgolic acid on the TNF-α, IL-6 and VCAM-1 level of ox-LDL treated cell lines. **(A)** TNF-α, **(B)** IL-6 and **(C)** VCAM-1. Data are presented as Mean ± SEM, *p < 0.05, compared to ox-LDL group.

**Figure 7** demonstrated the increased expression of IL-6, TNF-α, and VCAM-1 in the ox-LDL treated in hPBMCs. Ginkgolic acid treated group significantly (P < 0.05) reduced the expression of IL-6, TNF-α, and VCAM-1.

### Discussion

Studies suggest that the ox-LDL is the significant marker for identifying the cardiovascular disease (Lapointe et al., 2006; Brinkley et al., 2009). Ginkgolic acid may reduce oxidative stress and inflammation, both (oxidative stress and inflammation) concerned in enhancing the risk of cardiovascular diseases. Studies also suggest that the ginkgolic acid considerably reduced the adhesion, invasion, and migration of cancer cells. The current study scrutinized the beneficial effect of ginkgolic acid to reduce the NF-κB signaling pathway (Fukuda et al., 2009; Lee et al., 2014).

Previous research suggests that the MTT assay generally provide the information on the nature of noncytotoxicity cells *via* estimation of mitochondrial activity which is directly or indirectly concurrent to the cell viability, for both the attached and poorly attached cells (Park et al., 2006; Seidl and Zinkernagel, 2013). In the assay of MTT, the metabolically active cell decreases the MTT tetrazolium salt [3-(4,5-dimethylthiazol-2-yl)-2,5-diphenyltetrazolium bromide] and generates the crystal, whereas nonreactive cell or dead cells do not form the crystal. We estimated the cytotoxicity effect of ginkgolic acid on the HMEC-1 cells *via* performing the MTT assay. Cell viability assay and ginkgolic acid (20 µM) exhibit the nontoxic effect against the HMEC-1 cells.

Research suggests that the ox-LDL plays a significant role in the initiation and the expansion of oxidative stress and malonaldehyde (MDA) is the considerable marker of LPO involved in the breakdown of LPO (Tsuzura et al., 2004; Mitra et al., 2011a). HMEC-1 cells treated with ox-LDL exhibited the enhanced level of ROS and LPO product due to the formation of free radicals. Ginkgolic acid already confirmed their antioxidant nature because the hydroxyl group is found in the benzene ring system and eliminates the ROS (Negre-Salvayre et al., 2006; Mitra et al., 2011a). The current nature of ginkgolic acid may favor the reduction of ox-LDL. Ox-LDL significantly enhanced the intracellular ROS formation in HMEC-1 cells. Previous research suggests that ox-LDL is the strong inducer of ROS production. Ox-LDL was exhibited to boost the intracellular oxidation of DCFH dye, a process that has already been showed to depend on the production of intracellular ROS (Zapolska-Downar et al., 2002; Kim et al., 2010). Various free radicals, *viz*., hydrogen peroxide (H2O2), superoxide (·O_2_), NO, hydroxyl (·OH), peroxynitrite (·ONOO) radicals, generated the ROS in the endothelial cells (Re et al., 1999; Covas et al., 2006). In the current experimental study, we observed the increased level of ROS and ginkgolic acid treatment significantly reduced the formation of ROS *via* reducing the generation of free radicals. The result suggests the antioxidant nature of ginkgolic acid *via* scavenging the ROS.

Research suggests that the vascular endothelial injury leads to induce vascular disease in patients (Willerson, 2002; Ok et al., 2005). Ox-LDL is well known to trigger ECs to induce the production of chemotactic cytokines and adhesion molecules, attracting monocytes to the vascular wall for an inflammatory reaction (Yokoyama and Deckert, 1996; Selwyn et al., 1997). Theses mediators start the secretion of inflammatory cytokines that boost the migration and proliferation of smooth muscle cells resultant in atherosclerotic lesion development. The researcher focuses on their research to target the pro-inflammatory cytokines to scrutinize the protective effect of the drug. During atherosclerosis, pro-inflammatory cytokines such as TNF-α and IL-6 both have been related to vascular inflammation (Tedgui and Mallat, 2006; Papadimitraki and Boumpas, 2015). TNF-α has a synergistic effect on monocyte adhesion and endothelial dysfunction (Steyers and Miller, 2014). IL-6 increases the endothelial cell activation and induces the cell adhesion molecule expression such as VCAM-1, ICAM-1 and E-selectin on endothelial cells *via* trans-signaling (Ostrowski et al., 1999). Research suggests the level of pro-inflammatory cytokines such as TNF-α and IL-6 secrete during the HMEC-1 cells (Frostegård, 2005; Smith et al., 2012). The increased level of IL-6 in ox-LDL appears due to inducing the oxidative stress in the cell (Grivennikov and Karin, 2011). Boosting the level of IL-6, induce the inflammatory reaction and expand the cardiovascular disease. In our study, ginkgolic acid significantly reduced the level of IL-6 and suggesting the antioxidant and anti-inflammatory nature (Frostegård et al., 1999; Brüünsgaard and Pedersen, 2003). The level of TNF-α, up-regulated during the ox-LDL treatment could be due to endothelial apoptosis. Ginkgolic acid considerably reduced the TNF-α level and reduced the ox-LDL–induced endothelial apoptosis. Based on the result, we can conclude that ginkgolic acid considerably reduced the pro-inflammatory cytokines in ox-LDL treated HMEC-1 cells.

The researcher suggests that the prostaglandins are the bioactive signaling molecule isolated from the cyclooxygenase (COX) involved in the inflammatory reaction, especially in the circulation/regulation of the pro-inflammatory cytokines (Libby, 2002; Pirillo et al., 2013; Viola and Soehnlein, 2015). The continues generation of PGE_2_ was prominent in response to inflammatory stimuli. On the country, another inflammatory mediator such as 5-lipoxygenase (5-LOX) pathway mainly responsible for the fabrication of inflammatory lipid mediators having a significant role in the expansion of immunity along with it’s as pro-atherogenic agents (Kühn and O’Donnell, 2006; Wittwer and Hersberger, 2007; Pirillo et al., 2013). In the current experimental study, ginkgolic acid significantly down-regulated the LOX and PGE_2_ activity in ox-LDL treated cells and suggesting the anti-inflammatory effect. Various other signaling pathways such as a nitric oxide (NO) play a crucial role in the pathogenesis of inflammation. NO is synthesized *via* nitric oxide synthases (NOSs), which are present in the various tissues (Y. S., 2009). During the inflammation condition, endothelial nitric oxide synthase (eNOS) is responsible for the circulation of NO in the arteries and induced the inducible nitric oxide synthase (Förstermann and Münzel, 2006; Godo and Shimokawa, 2017). iNOS usually generates a higher amount of NO than eNOS and also plays a crucial role in the inflammation, apoptosis, and cellular damage. Previous research suggests that iNOS concurrently enhances O_2_ and NO production and nitrosative/oxidative stress in the atherosclerotic plaques (Cannon, 1998; Pistrosch et al., 2013). Our experimental study showed that the iNOS expression is enhanced in ox-LDL treated cells, which in turn signify the increase in NO production. The researcher suggests that the generated NO releases from the phagocytic and endothelial cells and reacts with the superoxide (O_2_) to produce the peroxynitrites (Forman and Torres, 2001). A similar effect was observed in our experimental study, ox-LDL showed the increased level of iNOS and NO and suggested the increased inflammatory reaction, and ginkgolic acid significantly reduced the NO and iNOS level, suggesting the anti-inflammatory effect.

NF-κB is a significant and ubiquitous transcription factor for genes that encrypt the cytokines including TNF-α and IL-6. Research suggests that the activation of NF-κB involves in the degradation of IκB protein (Alnemri et al., 2002). Phosphorylation of IκBα *via* cytokines, drugs viruses, and bacterial products quickly starts the degradation and translocation of NF-κB into the nucleus. After the activation of NF-κB, start the binding with the mRNAs expression and specific promoter elements of pro-inflammatory cytokines genes (Viatour et al., 2005; Yang et al., 2008). Research suggests that the ox-LDL activates the NF-κB in smooth muscle cells, endothelial cells, and fibroblasts (Mitra et al., 2011b; Mitra et al., 2011c). NF-κB also activates during the expansion of early atherosclerotic lesions. In the current experimental study, we exhibit that ox-LDL activates the nuclear translocation of NF-κB p65 subunit and upregulate the inflammatory reaction in cultured hPBMCs, and GA treatment downregulated the nuclear translocation of NF-κB p65 subunit and also decreased the inflammatory response (Morel et al., 2011; Mitra et al., 2011b). The current action, due to its antioxidant property, could be responsible for its anti-oxidant and anti-inflammatory effects as well because most of the pro-inflammatory genes are under the control of NF-κB signaling pathway, and GA can counter regulate these pathways.

## Conclusion

The current experimental study points toward the anti-inflammatory and antioxidant effect of ginkgolic acid against the ox-LDL–induced atherosclerosis. During the experimental study, GA played a preventive role against the ox-LDL–induced inflammation and oxidative stress in hPBMCs. The upregulation of pro-inflammatory cytokines in the ox-LDL group and translocation of NF- κB p65 subunit was observed to be downregulated *via* GA treatment. Furthermore, *in vivo* experimental investigation is warranted to explore the possible cardiovascular protective effect of ginkgolic acid in cardiovascular disease.

## Data Availability Statement

The raw data supporting the conclusions of this manuscript will be made available by the authors, without undue reservation, to any qualified researcher.

## Author Contributions

JZ and JY performed the experimental study. JZ and JY estimated the biochemical data. All authors equally contributed to proofreading.

## Conflict of Interest

The authors declare that the research was conducted in the absence of any commercial or financial relationships that could be construed as a potential conflict of interest.

## Abbreviations

AS, atherosclerosis; COX, cyclooxygenase; CVD, cardiovascular inflammation; DCFH-DA, dichlorodihydrofluorescein diacetate; DPPH, 2,2-diphenyl-1-picrylhydrazyl; HMEC-1, human microvascular endothelial cells; IL-1, interleukin-1; IL-2, interleukin-2; IL-6, interleukin-6 (IL-6); LDL, low lipid lipoproteins; LOX, lipoxygenase; LPO, lipid peroxidation; MDA, malondialdehyde; MMP, matrix metalloproteinases; MTT, 3-(4,5-dimethylthiazol-2-yl)-2,5-diphenyltetrazolium bromide; Myd88, myeloid differentiation factor 88; NF-κB, nuclear level, central transcriptional; NO, nitric oxide; nPBMCs, human peripheral blood mononuclear cells; ox-LDL, oxidized low-density lipoprotein; O2, superoxide; PGE2, prostaglandin E2; ROS, reactive oxygen species; TLR-4, toll-like receptor 4; TNF-α, tumor necrosis factor-α; VCAM-1, vascular cell adhesion protein 1.

## References

[B1] Adiels M. Olofsson S. O. Taskinen M. R. Borén J. (2008). Overproduction of very low-density lipoproteins is the hallmark of dyslipidemia in the metabolic syndrome. Arterioscler. Thromb. Vasc. Biol. 28 (7), 1225–1236 10.1161/ATVBAHA.107.160192 18565848

[B2] Akpolat M. Kanter M. Topcu-Tarladacalisir Y. Aydogdu N. (2011). Protective effect of flaxseed oil on renal injury in hyperlipidaemic rats: The effect of flaxseed oil on hyperlipidaemia. Phyther. Res. 25 (6), 796–802. 10.1002/ptr.3334 21077265

[B3] Alnemri E. S. Fernandes-Alnemri T. Lin J.-H. Poyet J.-L. Tsichlis P. N. Srinivasula S. M. (2002). Activation of the IκB Kinases by RIP via IKKγ/NEMO-mediated Oligomerization. J. Biol. Chem. 275, 37966–37977. 10.1074/jbc.M006643200 10980203

[B4] Aviram M. Rosenblat M. (2004). Paraoxonases 1, 2, and 3, oxidative stress, and macrophage foam cell formation during atherosclerosis development. Free Radic. Biol. Med. 1;37 (9), 1304–1316. 10.1016/j.freeradbiomed.2004.06.030 15454271

[B5] Boersma M. C. H. Dresselhaus E. C. De Biase L. M. Mihalas A. B. Bergles D. E. Meffert M. K. (2011). A Requirement for Nuclear Factor- B in Developmental and Plasticity-Associated Synaptogenesis. J. Neurosci. 6;31 (14), 5414–5425. 10.1523/JNEUROSCI.2456-10.2011 21471377 PMC3113725

[B6] Brinkley T. E. Nicklas B. J. Kanaya A. M. Satterfield S. Lakatta E. G. Simonsick E. M. (2009). Plasma oxidized low-density lipoprotein levels and arterial stiffness in older adults the health, aging, and body composition study. Hypertension. 53 (5), 846–852. 10.1161/HYPERTENSIONAHA.108.127043 19332658 PMC2692957

[B7] Brucker N. Moro A. M. Charão M. F. Durgante J. Freitas F. Baierle M. (2013). Biomarkers of occupational exposure to air pollution, inflammation and oxidative damage in taxi drivers. Sci. Total Environ. 1, 463–464, 884–893. 10.1016/j.scitotenv.2013.06.098 23872245

[B8] Brüünsgaard H. Pedersen B. K. (2003). Age-related inflammatory cytokines and disease. Immunol. Allergy Clin. North Am. 23 (1), 15–39. 10.1016/S0889-8561(02)00056-5 12645876

[B9] Burstein M. Scholnick H. R. Morfin R. (1970). Rapid method for the isolation of lipoproteins from human serum by precipitation with polyanions. J. Lipid Res. 11 (6), 583–595. 4100998

[B10] Cai H. Harrison D. G. (2000). Endothelial dysfunction in cardiovascular diseases: The role of oxidant stress. Circ. Res. 87, 840–844. 10.1161/01.RES.87.10.840 11073878

[B11] Cannon R. O. (1998). Role of nitric oxide in cardiovascular disease: Focus on the endothelium. Clin. Chem. 44 (8 Pt 2), 1809–1819. 9702990

[B12] Cossarizza A. Baccarani-Contri M. Kalashnikova G. Franceschi C. (1993). A new method for the cytofluorometric analysis of mitochondrial membrane potential using the J-aggregate forming lipophilic cation 5,5’,6,6’-tetrachloro-1,1’,3,3’-tetraethylbenzim idazolcarbocyanine iodide (JC-1). Biochem. Biophys. Res. Commun. 30;197 (1), 40–45. 10.1006/bbrc.1993.2438 8250945

[B13] Covas M. I. De La Torre K. Farré-Albaladejo M. Kaikkonen J. Fitó M. López-Sabater C. (2006). Postprandial LDL phenolic content and LDL oxidation are modulated by olive oil phenolic compounds in humans. Free Radic. Biol. Med. 608–616. 10.1016/j.freeradbiomed.2005.09.027 16458191

[B14] Dichtl W. Nilsson L. Goncalves I. Ares M. P. S. Banfi C. Calara F. (1999). Very low-density lipoprotein activates nuclear factor-κB in endothelial cells. Circ. Res. 84, 1085–1094. 10.1161/01.RES.84.9.1085 10325246

[B15] Ding Z. Liu S. Wang X. Khaidakov M. Dai Y. Mehta J. L. (2013). Oxidant stress in mitochondrial DNA damage, autophagy and inflammation in atherosclerosis. Sci. Rep. 3, 1077. 10.1038/srep01077 23326634 PMC3546319

[B16] Esper R. Vilariño J. Machado R. Paragano A. (2008). Endothelial dysfunction in normal and abnormal glucose metabolism. Adv. Cardiol. 45, 17–43. 10.1159/000115120 18230954

[B17] Ferretti G. Bacchetti T. Masciangelo S. Bicchiega V. (2010). Effect of phytosterols on copper lipid peroxidation of human low-density lipoproteins. Nutrition. 26 (3), 296–304. 10.1016/j.nut.2009.04.015 19815390

[B18] Forman H. J. Torres M. (2001). Redox signaling in macrophages. Mol. Aspects Med. 22 (4–5), 189–216. 10.1016/S0098-2997(01)00010-3 11679166

[B19] Förstermann U. Münzel T. (2006). Endothelial nitric oxide synthase in vascular disease: From marvel to menace. Circulation. 4;113 (13), 1708–1714. 10.1161/CIRCULATIONAHA.105.602532 16585403

[B20] Friedewald W. T. Levy R. I. Fredrickson D. S. (1972). Estimation of the concentration of low-density lipoprotein cholesterol in plasma, without the use of the preparative ultracentrifuge. Clin. Chem. 18 (6), 499–502. 4337382

[B21] Frostegård J. (2005). SLE, atherosclerosis and cardiovascular disease. J. Intern. Med. 257 (6) 485–495. 10.1111/j.1365-2796.2005.01502.x 15910552

[B22] Frostegård J. Ulfgren A. K. Nyberg P. Hedin U. Swedenborg J. Andersson U. (1999). Cytokine expression in advanced human atherosclerotic plaques: Dominance of pro-inflammatory (Th1) and macrophage-stimulating cytokines. Atherosclerosis. 145 (1), 33–43. 10.1016/S0021-9150(99)00011-8 10428293

[B23] Fukuda I. Ito A. Hirai G. Nishimura S. Kawasaki H. Saitoh H. (2009). Ginkgolic Acid Inhibits Protein SUMOylation by Blocking Formation of the E1-SUMO Intermediate. Chem. Biol. 27;16 (2), 133–140. 10.1016/j.chembiol.2009.01.009 19246003

[B24] Geng H. Wang A. Rong G. Zhu B. Deng Y. Chen J. (2010). The effects of ox-LDL in humanatherosclerosis may be mediated in part via the toll-like receptor 4 pathway. Mol. Cell. Biochem. 342 (1–2), 201–206. 10.1007/s11010-010-0484-8 20467793

[B25] Gharavi N. M. Gargalovic P. S. Chang I. Araujo J. A. Clark M. J. Szeto W. L. (2007). High-density lipoprotein modulates oxidized phospholipid signaling in human endothelial cells from proinflammatory to anti-inflammatory. Arterioscler. Thromb. Vasc. Biol. 27, 1346–1353. 10.1161/ATVBAHA.107.141283 17379837

[B26] Gimbrone M. A. García-Cardeña G. (2016). Endothelial Cell Dysfunction and the Pathobiology of Atherosclerosis. Circ. Res. 19;118 (4), 620–636. 10.1161/CIRCRESAHA.115.306301 26892962 PMC4762052

[B27] Godo S. Shimokawa H. (2017). Endothelial Functions. Arterioscler. Thromb. Vasc. Biol. 37, e108–e114. 10.1161/ATVBAHA.117.309813 28835487

[B28] Gritters M. Grooteman M. P. C. Schoorl M. Schoorl M. Bartels P. C. M. Scheffer P. G. (2006). Citrate anticoagulation abolishes degranulation of polymorphonuclear cells and platelets and reduces oxidative stress during haemodialysis. Nephrol. Dial. Transplant. 21 (1), 153–159. 10.1093/ndt/gfi069 16144857

[B29] Grivennikov S. I. Karin M. (2011). Inflammatory cytokines in cancer: Tumour necrosis factor and interleukin 6 take the stage. Ann. Rheum. Dis. 70 1, 104–108. 10.1136/ard.2010.140145 21339211

[B30] Hadi H. A. R. Carr C. S. Al Suwaidi J. (2005). Endothelial dysfunction: cardiovascular risk factors, therapy, and outcome. Vasc. Health Risk Manag. 1 (3), 183–98. PMC1993955 17319104

[B31] Hamilton C. A. (1997). Low-Density Lipoprotein and Oxidised Low-Density Lipoprotein: Their role in the development of atherosclerosis. Pharmacol. Ther. 10.1016/S0163-7258(96)00202-1 9336016

[B32] Han C. Y. Kargi A. Y. Omer M. Chan C. K. Wabitsch M. O’Brien K. D. (2010). Differential effect of saturated and unsaturated free fatty acids on the generation of monocyte adhesion and chemotactic factors by adipocytes: Dissociation of adipocyte hypertrophy from inflammation. Diabetes. 59 (2), 386-396. 10.2337/db09-0925 19934003 PMC2809975

[B33] Han M. Song Y. Zhang X. (2016). Quercetin suppresses the migration and invasion in human colon cancer Caco-2 cells through regulating toll-like receptor 4/nuclear factor-kappa B pathway. Pharmacogn. Mag. 12 (2), S237–S244. 10.4103/0973-1296.179654 27279714 PMC4883086

[B34] Hartge M. M. Unger T. Kintscher U. (2007). The endothelium and vascular inflammation in diabetes. Diabetes Vasc. Dis. Res. 4 (2) 84–88. 10.3132/dvdr.2007.025 17654441

[B35] Higashi Y. Noma K. Yoshizumi M. Kihara Y. (2009). Endothelial Function and Oxidative Stress in Cardiovascular Diseases. Circ. J. 41 (10), 607–609. 10.1253/circj.CJ-08-1102 19194043

[B36] Hlaing T. T. Park A. (2013). Hyperlipidaemia. Medicine (Baltimore). 41 (10), 607–609. 10.1016/j.mpmed.2013.07.004

[B37] Ho K. J. (2018). “Cardiovascular diseases,” in Nutritional Aspects of Aging, (Boca Raton Imprint CRC Press) 257. 10.1201/9781351075145

[B38] Holligan S. D. Berryman C. E. Wang L. Flock M. R. Harris K. A. Kris-Etherton P. M. (2012). “Atherosclerotic Cardiovascular Disease,” in Present Knowledge in Nutrition, Tenth Edition. (Wiley Online Library). 10.1002/9781119946045.ch48

[B39] Jonas A. Phillips M. C. (2008). “Lipoprotein structure,” in Biochemistry of Lipids, Lipoproteins and Membranes. 485−506. 10.1016/B978-044453219-0.50019-2

[B40] Kim J. A. Kong C. S. Kim S. K. (2010). Effect of Sargassum thunbergii on ROS mediated oxidative damage and identification of polyunsaturated fatty acid components. Food Chem. Toxicol. 48 (5), 1243–129. 10.1016/j.fct.2010.02.017 20171254

[B41] Kubes P. Suzuki M. Granger D. N. (1991). Nitric oxide: An endogenous modulator of leukocyte adhesion (inflammation/shear rate/NG-monomethyl-L-arginine/NG-nitro-L-arginine methyl ester/arginine). 88 (11), 4651–4655. 10.1073/pnas.88.11.4651 PMC51723 1675786

[B42] Kühn H. O’Donnell V. B. (2006). Inflammation and immune regulation by 12/15-lipoxygenases. Prog. Lipid Res. 45 (4), 334–356. 10.1016/j.plipres.2006.02.003 16678271

[B43] Kumar V. Al-Abbasi F. A. Ahmed D. Verma A. Mujeeb M. Anwar F. (2015). Paederia foetida Linn. inhibits adjuvant induced arthritis by suppression of PGE < inf > 2 and COX-2 expression via nuclear factor-κB. Food Funct. 6 (5), 1652–1666. 10.1039/C5FO00178A 25893742

[B44] Lamon B. D. Hajjar D. P. (2008). Inflammation at the molecular interface of atherogenesis: An Anthropological Journey. Am. J. Pathol. 173 (5), 1253-1264. 10.2353/ajpath.2008.080442 18948435 PMC2570117

[B45] Lapointe A. Couillard C. Lemieux S. (2006). Effects of dietary factors on oxidation of low-density lipoprotein particles. J. Nutr. Biochem. 17 (10), 645–658. 10.1016/j.jnutbio.2006.01.001 16517144

[B46] Lee J. H. Kim Y. G. Ryu S. Y. Cho M. H. Lee J. (2014). Ginkgolic acids and Ginkg o biloba extract inhibit Escherichia coli O157: H7 and Staphylococcus aureus biofilm formation. Int. J. Food Microbiol. 17;174, 47–55. 10.1016/j.ijfoodmicro.2013.12.030 24457153

[B47] Lerner C. A. Sundar I. K. Yao H. Gerloff J. Ossip D. J. McIntosh S. (2015). Vapors produced by electronic cigarettes and E-juices with flavorings induce toxicity, oxidative stress, and inflammatory response in lung epithelial cells and in mouse lung. PLoS One. 6;10 (2), e0116732. 10.1371/journal.pone.0116732 25658421 PMC4319729

[B48] Libby P. (2002). Inflammation in atherosclerosis. Nature. 19–26;420 (6917), 868–874. 10.1038/nature01323 12490960

[B49] Lin W. C. Deng J. S. Huang S. S. Wu S. H. Chen C. C. Lin W. R. (2017). Anti-inflammatory activity of Sanghuangporus sanghuang mycelium. Int. J. Mol. Sci. 7;18 (2), pii: . 10.3390/ijms18020347 PMC5343882 28178212

[B50] Lluís L. Taltavull N. Muñoz-Cortés M. Sánchez-Martos V. Romeu M. Giralt M. (2013). Protective effect of the omega-3 polyunsaturated fatty acids: Eicosapentaenoic acid/Docosahexaenoic acid 1:1 ratio on cardiovascular disease risk markers in rats. Lipids Health Dis. 1;12, 140. 10.1186/1476-511X-12-140 24083393 PMC3850782

[B51] Lu J. Mitra S. Wang X. Khaidakov M. Mehta J. L. (2011). Oxidative Stress and Lectin-Like Ox-LDL-Receptor LOX-1 in Atherogenesis and Tumorigenesis. Antioxid. Redox. Signal. 15 (8), 2301–2333. 10.1089/ars.2010.3792 21338316

[B52] Macho A. Decaudin D. Castedo M. Hirsch T. Susin S. A. Zamzami N. (1996). Chloromethyl-X-rosamine is an aldehyde-fixable potential-sensitive fluorochrome for the detection of early apoptosis. Cytometry. 25 (4), 333–340. 10.1002/(SICI)1097-0320(19961201)25:4<333::AID-CYTO4>3.0.CO;2-E 8946140

[B53] Mendis S. Puska P. Norrving B. (2011). Global atlas on cardiovascular disease prevention and control. World Heal. Organ.

[B54] Mitra S. Deshmukh A. Sachdeva R. Lu J. Mehta J. L. (2011a). Oxidized low-density lipoprotein and atherosclerosis implications in antioxidant therapy. Am. J. Med. Sci. 342 (2), 135–142. 10.1097/MAJ.0b013e318224a147 21747278

[B55] Mitra S. Deshmukh A. Sachdeva R. Lu J. Mehta J. L. (2011b). Oxidized low-density lipoprotein and atherosclerosis implications in antioxidant therapy. Am. J. Med. Sci. 342 (2), 135–142. 10.1097/MAJ.0b013e318224a147 21747278

[B56] Mitra S. Goyal T. Mehta J. L. (2011c). Oxidized LDL, LOX-1 and atherosclerosis. Cardiovasc. Drugs Ther. 25 (5), 419–429. 10.1007/s10557-011-6341-5 21947818

[B57] Morel D. W. DiCorleto P. E. Chisolm G. M. (2011). Endothelial and smooth muscle cells alter low density lipoprotein in vitro by free radical oxidation. Arterioscler. An Off. J. Am. Hear. Assoc. Inc. 4 (4), 357–364. 10.1161/01.ATV.4.4.357 6466193

[B58] Negre-Salvayre A. Dousset N. Ferretti G. Bacchetti T. Curatola G. Salvayre R. (2006). Antioxidant and cytoprotective properties of high-density lipoproteins in vascular cells. Free Radic. Biol. Med. 41 (7), 1031–1040. 10.1016/j.freeradbiomed.2006.07.006 16962927

[B59] Ok E. Basnakian A. G. Apostolov E. O. Barri Y. M. Shah S. V. (2005). Carbamylated low-density lipoprotein induces death of endothelial cells: A link to atherosclerosis in patients with kidney disease. Kidney Int. 68 (1), 173–178. 10.1111/j.1523-1755.2005.00391.x 15954906

[B60] Ostrowski K. Rohde T. Asp S. Schjerling P. Pedersen B. K. (1999). Pro- and anti-inflammatory cytokine balance in strenuous exercise in humans. J. Physiol. 10.1111/j.1469-7793.1999.287ad.x PMC2269132 9925898

[B61] Papadimitraki E. D. Boumpas D. T. (2015). “Inflammation and atherosclerosis,” in Introduction to Translational Cardiovascular Research. 217–238. 10.1007/978-3-319-08798-6_13

[B62] Park H. J. Zhang Y. Georgescu S. P. Johnson K. L. Kong D. Galper J. B. (2006). Human umbilical vein endothelial cells and human dermal microvascular endothelial cells offer new insights into the relationship between lipid metabolism and angiogenesis. Stem Cell Rev. 2 (2), 93–102. 10.1385/SCR:2:2:93 17237547

[B63] Pirillo A. Norata G. D. Catapano A. L. (2013). LOX-1, OxLDL, and Atherosclerosis. Mediators Inflamm. 2013, 152786. 10.1155/2013/152786 23935243 PMC3723318

[B64] Pistrosch F. Schaper F. Hanefeld M. (2013). “The metabolic syndrome and cardiovascular disease,” in The Metabolic Syndrome: Pharmacology and Clinical Aspects. 11 (3), 155–61. 10.1007/978-3-7091-1331-8_4

[B65] Potenza M. Gagliardi S. Nacci C. Carratu M. Montagnani M. (2008). Endothelial Dysfunction in Diabetes: From Mechanisms to Therapeutic Targets. Curr. Med. Chem. 16 (1), 94–112. 10.2174/092986709787002853 19149564

[B66] Rao A. V. (2002). Lycopene, tomatoes, and the prevention of coronary heart disease. Exp. Biol. Med. (Maywood). 227 (10), 908–13. 10.1177/153537020222701011 12424333

[B67] Re R. Pellegrini N. Proteggente A. Pannala A. Yang M. Rice-Evans C. (1999). Antioxidant activity applying an improved ABTS radical cation decolorization assay. Free Radic. Biol. Med. 26 (9–10), 1231–1237. 10.1016/S0891-5849(98)00315-3 10381194

[B68] Reamy B. V. Williams P. M. Kuckel D. P. (2018). Prevention of Cardiovascular Disease. Prim. Care Clin. Off. Pract. 45 (1), 25–44. 10.1016/j.pop.2017.11.003 29406943

[B69] Reis J. P. Lutsey P. L. (2012). “Vitamin D and cardiovascular disease,” in Vitamin D: Oxidative Stress, Immunity, and Aging. 10.1201/b13714

[B70] Rocha D. M. Caldas A. P. Oliveira L. L. Bressan J. Hermsdorff H. H. (2016). Saturated fatty acids trigger TLR4-mediated inflammatory response. Atherosclerosis. 244, 211–5. 10.1016/j.atherosclerosis.2015.11.015 26687466

[B71] Ross R. (1999). Inflammation or Atherogenesis. N. Engl. J. Med. 340, 115–126. 10.1056/NEJM199901143400207 9887164

[B72] Schleicher E. Friess U. (2007). Kidney Int. (106), S17–26). 10.1038/sj.ki.5002382 17653206

[B73] Seidl K. Zinkernagel A. S. (2013). The MTT assay is a rapid and reliable quantitative method to assess Staphylococcus aureus induced endothelial cell damage. J. Microbiol. Methods. 92 (3), 307–309. 10.1016/j.mimet.2012.12.018 23275136

[B74] Selwyn A. P. Kinlay S. Libby P. Ganz P. (1997). Atherogenic lipids, vascular dysfunction, and clinical signs of ischemic heart disease. Circulation. 95, 5–7. 10.1161/01.CIR.95.1.5 8994406

[B75] Slager C. J. Wentzel J. J. Gijsen F. J. H. Schuurbiers J. C. H. van der Wal A. C. van der Steen A. F. W. (2005). The role of shear stress in the generation of rupture-prone vulnerable plaques. Nat. Clin. Pract. Cardiovasc. Med. 2 (8), 401–407. 10.1038/ncpcardio0274 16119702

[B76] Smith J. A. Das A. Ray S. K. Banik N. L. (2012). Role of pro-inflammatory cytokines released from microglia in neurodegenerative diseases. Brain Res. Bull. 4;87 (1), 10–20. 10.1016/j.brainresbull.2011.10.004 22024597 PMC9827422

[B77] Steyers C. M. Miller F. J. (2014). Endothelial dysfunction in chronic inflammatory diseases. Int. J. Mol. Sci. 15 (7), 11324–11349. 10.3390/ijms150711324 24968272 PMC4139785

[B78] Suematsu M. Suzuki H. Delano F. A. Schmid-Schönbein G. W. (2002). The inflammatory aspect of the microcirculation in hypertension: Oxidative stress, leukocytes/endothelial interaction, apoptosis. Microcirculation. 9 (4), 259–276. 10.1038/sj.mn.7800141 12152103

[B79] Tedgui A. Mallat Z. (2006). Cytokines in Atherosclerosis: Pathogenic and Regulatory Pathways. Physiol. Rev. 86 (2), 515–81. 10.1152/physrev.00024.2005 16601268

[B80] Troosters T. (2012). “Cardiovascular disease,” in Chronic Obstructive Pulmonary Disease: Co-Morbidities and Systemic Consequences. 10.1007/978-1-60761-673-3_4

[B81] Tsuzura S. Ikeda Y. Suehiro T. Ota K. Osaki F. Arii K. (2004). Correlation of plasma oxidized low-density lipoprotein levels to vascular complications and human serum paraoxonase in patients with type 2 diabetes. Metabolism. 53 (3), 297–302. 10.1016/j.metabol.2003.10.009 15015140

[B82] Vanhoutte P. M. Shimokawa H. Feletou M. Tang E. H. C. (2017). Endothelial dysfunction and vascular disease – a 30th anniversary update. Acta Physiol. 219 (1), 22–96. 10.1111/apha.12646 26706498

[B83] Vanhoutte P. M. Shimokawa H. Tang E. H. C. Feletou M. (2009). Endothelial dysfunction and vascular disease. Acta Physiol. 196 (2), 193–222. 10.1111/j.1748-1716.2009.01964.x 19220204

[B84] Viatour P. Merville M. P. Bours V. Chariot A. (2005). Phosphorylation of NF-κB and IκB proteins: Implications in cancer and inflammation. Trends Biochem. Sci. 30 (1), 43–52. 10.1016/j.tibs.2004.11.009 15653325

[B85] Viola J. Soehnlein O. (2015). Atherosclerosis - A matter of unresolved inflammation. Semin. Immunol. 27 (3), 184–93. 10.1016/j.smim.2015.03.013 25865626

[B86] Willerson J. T. (2002). Systemic and local inflammation in patients with unstable atherosclerotic plaques. Prog. Cardiovasc. Dis. 44 (6), 469–478. 10.1053/pcad.2002.123782 12077720

[B87] Wiseman H. O’Reilly J. D. Adlercreutz H. Mallet A. I. Bowey E. A. Rowland I. R. (2000). Isoflavone phytoestrogens consumed in soy decrease F2-isoprostane concentrations and increase resistance of low-density lipoprotein to oxidation in humans. Am. J. Clin. Nutr. 72 (2), 395–400. 10.1093/ajcn/72.2.395 10919933

[B88] Wittwer J. Hersberger M. (2007). The two faces of the 15-lipoxygenase in atherosclerosis. Prostaglandins Leukot. Essent. Fat. Acids. 77 (2), 67–77. 10.1016/j.plefa.2007.08.001 17869078

[B89] Y. S. K. (2009). Anti-inflammatory therapy in patients with acute respiratory distress syndrome. Respirology. Suppl 3, A93–295. 10.1111/j.1440-1843.2009.01656.x

[B90] Yan X. Lee S. Gugiu B. G. Koroniak L. Jung M. E. Berliner J. (2014). Fatty acid epoxyisoprostane E2 stimulates an oxidative stress response in endothelial cells. Biochem. Biophys. Res. Commun. 444 (1), 69–74. 10.1016/j.bbrc.2014.01.016 24434148 PMC3962750

[B91] Yang W. S. Seo J. W. Han N. J. Choi J. Lee K.-U. Ahn H. (2008). High glucose-induced NF-κB activation occurs via tyrosine phosphorylation of IκBα in human glomerular endothelial cells: involvement of Syk tyrosine kinase. Am. J. Physiol. Physiol. 294 (5), F1065–F1075. 10.1152/ajprenal.00381.2007 18353872

[B92] Yokoyama H. Deckert T. (1996). Central role of TGF-β in the pathogenesis of diabetic nephropathy and macrovascular complications: A hypothesis. Diabet. Med. 13 (4), 313–320. 10.1002/(SICI)1096-9136(199604)13:4<313::AID-DIA56>3.0.CO;2-7 9162605

[B93] Yu M. Kang X. Xue H. Yin H. (2011). Toll-like receptor 4 is up-regulated by mTOR activation during THP-1 macrophage foam cells formation. Acta Biochim. Biophys. Sin. (Shanghai). 43 (12), 940–947. 10.1093/abbs/gmr093 22015781

[B94] Yu S. Wang X. He X. Wang Y. Gao S. Ren L. (2016). Curcumin exerts anti-inflammatory and antioxidative properties in 1-methyl-4-phenylpyridinium ion (MPP+)-stimulated mesencephalic astrocytes by interference with TLR4 and downstream signaling pathway. Cell Stress Chaperones. 21 (4), 697–705. 10.1007/s12192-016-0695-3 27164829 PMC4908001

[B95] Yung L. Leung F. Yao X. Chen Z.-Y. Huang Y. (2012). Reactive Oxygen Species in Vascular Wall. Cardiovasc. Hematol. Disord. Targets. (6), 1–19. 10.2174/187152906776092659 16724932

[B96] Zapolska-Downar D. Zapolski-Downar A. Naruszewicz M. Siennicka A. Krasnodbska B. Kolodziej B. (2002). Protective properties of artichoke (Cynara scolymus) against oxidative stress induced in cultured endothelial cells and monocytes. Life Sci. 71 (24), 2897–2908. 10.1016/S0024-3205(02)02136-7 12377270

